# Pets for pediatric transplant recipients: To have or not to have

**DOI:** 10.3389/fvets.2022.974665

**Published:** 2022-09-08

**Authors:** Lucía Platero, Paula Garcia-Sanchez, Talía Sainz, Cristina Calvo, Irene Iglesias, Fernando Esperon, Ricardo de la Fuente, Esteban Frauca, Antonio Perez-Martinez, Ana Mendez-Echevarria

**Affiliations:** ^1^Faculty of Medicine, Autonomous University of Madrid, Madrid, Spain; ^2^Pediatric Emergency Department, La Paz University Hospital, Madrid, Spain; ^3^University Hospital La Paz Research Institute (IdiPAZ), Madrid, Spain; ^4^Centro de Investigación en Red en Enfermedades Infecciosas (CIBERINFEC), Instituto de Salud Carlos III, Madrid, Spain; ^5^Pediatric Infectious Disease Department, La Paz University Hospital, Madrid, Spain; ^6^ERN Transplant Child, Madrid, Spain; ^7^Center for Animal Health Research (CISA), Madrid, Spain; ^8^Veterinary Faculty, European University of Madrid, Madrid, Spain; ^9^Department of Animal Health, Veterinary Faculty, Complutense University, Madrid, Spain; ^10^Pediatric Hepatology Department, La Paz University Hospital, Madrid, Spain; ^11^Pediatric Hemato-Oncology Department, La Paz University Hospital, Madrid, Spain

**Keywords:** pets, transplant, zoonosis, children, survey

## Abstract

Pets have many health, emotional and social benefits for children, but the risk of zoonotic infections cannot be underestimated, especially for immunosuppressed patients. We report the recommendations given by health professionals working with pediatric transplant recipients to their families regarding pet ownership. An online survey addressing zoonosis knowledge and recommendations provided by health care practitioners regarding pets was distributed to clinicians treating pediatric transplant recipients. The European Society of Pediatric Infectious Disease (ESPID) and the European Reference Network ERN-TransplantChild, which works to improve the quality of life of transplanted children, allowed the online distribution of the survey. A total of 151 practitioners from 28 countries participated in the survey. Up to 29% of the respondents had treated at least one case of zoonosis. Overall, 58% of the respondents considered that the current available evidence regarding zoonotic risk for transplanted children of having a pet is too scarce. In addition, up to 23% of the surveyed professionals recognized to be unaware or outdated. Still, 27% of the respondents would advise against buying a pet. Practitioners already owning a pet less frequently advised patients against pet ownership, whereas non-pet-owners were more keen to advise against pet ownership (*p* = 0.058). 61% of the participants stated that there were no institutional recommendations regarding pets in their centers/units. However, 43% of them reported therapeutic initiatives that involved animals in their centers. Infectious disease specialists were more likely to identify zoonotic agents among a list of pathogens compared to other specialists (*p* < 0.05). We have observed a huge heterogeneity among the recommendations that health care providers offer to families in terms of risk related to pet ownership for transplant recipients. The lack of evidence regarding these recommendations results in practitioners' recommendations based on personal experience.

## Introduction

About 88 million households in the European Union own a pet (1), with rates of household penetration for pet-ownership ranging from 38 to 68% in the European Union and U.S.A. (1, 2). Animal-assisted therapies (AAT) have also been increasingly promoted in recent years, both outside and within health centers (3). Animal contact is known to have beneficial psychological and physical effects, stimulating emotional well-being, physical activity and more time spent outdoors (4). Children who have pets present emotional disorders less frequently than those without pets (5) and patients visited by animals reported decreased anxiety levels compared with patients who receive visits only from volunteers (6). The presence of a dog during venipuncture procedures reduces pain and distress in children (7).

However, animal contact also entails certain risks. Bites and scratches are not uncommon, patients might develop allergies and contact with animals entails risk of zoonoses. In the particular case of immunocompromised patients, opportunistic infections can lead to fatal outcomes. This is especially relevant for children, which are at higher risk of zoonosis due to their close contact with animals and behavioral habits.

However, families facing the diagnosis of a chronic disease in one of their children often acquire pets in an attempt to provide emotional support for the patient, and to increase the quality of life of that child (8). These include pediatric transplant recipients as well. Most guidelines on healthy lifestyles for transplant recipients include recommendations regarding pet ownership. However, these are mostly based on experts' opinion or on case reports, without providing the strength of the recommendations and the quality of evidence grade (9), or provide poor quality and strength regarding most recommendations (10). The decision is therefore left to the discretion of the attending healthcare practitioner (9, 11–13). The lack of evidence translates into great heterogeneity in clinical practice, in a context characterized by an increasing numbers of transplant recipients, along with a generalization of AATs.

A study in the United Kingdom found that only 4 out of 20 pediatric oncology centers offered specific recommendations regarding contact with animals (13). This lack of evidence-based recommendations could lead to unexpected zoonotic exposures. In fact, high-risk exposures (such a as bed sharing or face licking) have been reported in hemato-oncology patients who own pets (14). Potentially zoonotic pathogens were common among asymptomatic dogs and cats that took part in a university-based AAT program, and among pets living with immunocompromised children (15, 16).

To address the risk perception of health care practitioners in terms of pet ownership, and to analyze the heterogeneity and determinants of professional recommendations, a survey was conducted among healthcare practitioners working in the field of pediatric transplantation in different countries.

## Methods

We conducted an international, observational, cross-sectional study among health care providers involved in pediatric transplant. An anonymous questionnaire designed by pediatric infectious disease specialists and veterinarians was distributed online by the participating scientific societies (Supplementary File 1). The European Reference Network ERN-TransplantChild approved the distribution of the questionnaire among 124 professionals working in 40 healthcare centers from the European Network, between June and December 2020. The surveyed professionals were allowed to send the survey to other colleagues working in pediatric transplantation in their center. During the same period, the survey was also online distributed through the European Society for Pediatric Infectious Diseases to all its members, although only those specialists attending transplant patients were asked to answer it. The survey was sent twice during the 6-month period.

The questionnaire included questions addressing risk perception, knowledge of transmission routes, screening for zoonosis in routine clinical practice and general recommendations provided by the professionals and their institutions. Epidemiological data were collected simultaneously to identify the determinants of recommendations, such as years of experience, previous experience treating zoonosis or pet ownership. The risk awareness was stratified by clinical specialties to address the potential contribution of infectious disease specialist. The study was approved by the Ethics Committee of Hospital La Paz (PI-4770). Qualitative data were expressed as absolute frequencies and/or percentages; quantitative data were expressed as either medians and interquartile ranges (IQR), ranges, or means and standard deviations, depending on data distribution. We used chi-squared test and Fisher's exact test for categorical variables, and Student's *t*-test or non-parametric tests as appropriate for the continuous variables. A two-sided value of *p* ≤ 0.05 was considered statistically significant. The statistical analysis was performed using Stata v17.0 (StataCorp LP, College Station, TX, USA) and Prism v.7.0 (GraphPad, Inc., La Jolla, CA, USA).

## Results

### Characteristics of the survey respondents

A total of 151 healthcare practitioners from 78 hospitals in 28 countries participated in the survey, of which 79% were European (106/134) (Supplementary File 2). Table 1 summarizes the main characteristics of the study participants, who were mainly pediatricians treating pediatric solid-organ transplant recipients, hematologists specializing in hematopoietic stem cell transplantation, and pediatric infectious disease specialists. Over 47% of the respondents had up to 15 years of experience in transplantation and worked in units treating up to 100 pediatric transplant recipients.

**Table 1:** Demographic characteristics of the respondents.

**Characteristic**		**Total** **(*N* = 151)**	**%**
Gender	Male	58	39.19
	Female	90	60.81
	Non-responders	3	
Age, years	<35	11	7.38
	36–45	43	28.86
	46–55	50	33.56
	>55	45	30.20
	Non-responders	2	
Region	Europe	106	79.10
	North America	17	12.69
	South America	5	3.73
	Africa	2	1.49
	Asia	2	1.49
	Oceania	2	1.49
	Non-responders	17	
Specialty	Pediatrician working in SOT^(1)^	57	38.26
	Hematologist working in HSCT^(2)^	46	30.87
	Infectious diseases	37	24.83
	Surgeon/Others	9	6.04
	Non-responders	2	
Type of transplant	HSCT^(2)^	58	39.73
recipient treated by	Liver	15	10.27
the practitioners	Heart	9	6.17
	Kidney	19	13.01
	Lung	3	2.05
	Multivisceral/several organs	42	28.77
	Non-responders	5	
Experience, years	<5	26	17.45
	5–10	28	18.79
	10–15	25	16.78
	>15	70	46.98
	Non-responders	2	
Number of children	0–25	21	14.09
followed-up in their	25–50	33	22.15
units/departments	50–75	12	8.05
	75–100	12	8.05
	>100	71	47.65
	Non-responders	2	
Pet ownership	Has pet(s)	60	40
	Does not have a pet(s)	90	60
	Non-responders	1	

(1) Solid organ transplant,

(2) Hematopoietic stem cell transplant.

A total of 69% (93/151) of the participants acknowledged that they do not provide specific recommendations regarding pets and animal contact to their patients. However, 42.6% (64/150) worked in units or centers in which initiatives involving animals were ongoing. Excluding the responses of the pediatric infectious disease specialists, 33.9% (38/112) of the respondents stated that their transplantation team did not include an infectious disease specialist.

### Clinical practice and recommendations regarding pets

Up to 23.3% (34/146) of study participants acknowledged to be unaware of current pet ownership recommendations, while 58.2% (85/146) of the respondents considered the evidence insufficient as to lead to evidence-based recommendations. Among the participants working in units/centers with no specific recommendations regarding pets, only 58.2% (53/91) actively asked about pet ownership during the anamnesis, and 67.5% (102/151) acknowledged to be unaware of the rate of pet ownership among their patients.

**Figure 1:**
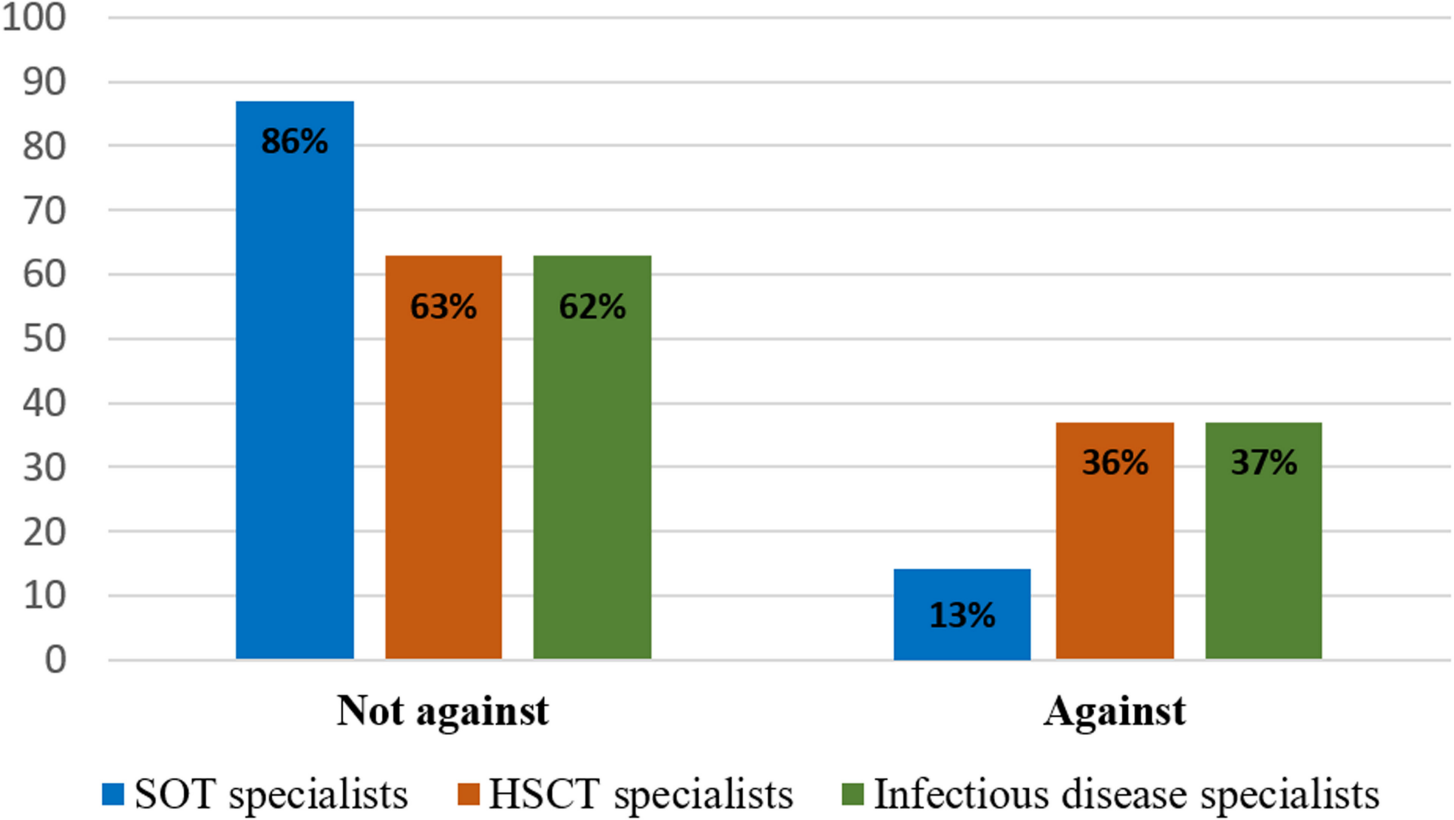
Recommendations given regarding pet ownership according to the various specialists.

In terms of the recommendations provided to families, 27% (40/149) of the respondents would recommend against buying a new pet. However, in cases where the patient already owns a pet, only 9% (13/149) of the respondents would advise against, while 78% (116/149) would recommend keeping the pet, with no significant differences between the various specialist groups. The hematopoietic stem cell transplantation specialists and the infectious disease specialists advised against pet ownership more frequently than the solid organ transplantation specialists (*p* = 0.02; Figure 1). Health care practitioners owning pets were less likely to advise against pet ownership [40% (60/150) vs. 60% (90/150); *p* = 0.058].

Regarding the perception of risk for various types of pets, we compared the respondents' answers to the published guidelines (9–13). These guidelines discourage patients to own reptiles and turtles. However, one out of three responders believed that there are no health risks in having turtles in the household, and one out of four responders for reptiles (Table 2).

**Table 2:** The risks imposed by pet ownership perceived by the 151 respondents.

**Animal**	**High Risk, %**	**Low Risk/** **No Risk, %**	**No Answer /** **Doesn't Know, %**	**Risk Considered in Guidelines (5–9)**
Dog*	18.54	74.84	6.62	Low (high in the case of puppies)
Fish	12.58	72.85	14.57	Low
Cat*	39.74	52.98	7.28	Medium (high in the case of kittens)
Bird^+^/chicks^++^	58.94	32.45	8.61	Medium
Rabbit^†^/Rodents^‡^	36.42	49.01	14.57	Medium
Turtle	46.36	**33.11**	20.53	**High**
Reptile	49.67	**26.49**	23.84	**High**

* Dogs and cats: Younger than 6 months of age should be avoided; Outdoor dogs and cats associated higher risk of zoonoses.

+ Birds: Risk of psittacosis and cryptococcosis, which is particularly high for lung transplant recipients.

++ Chicks and ducklings: High risk of Salmonella/Campylobacter transmission.

† Rabbits: Risk of Salmonella and Microsporidia.

‡ Rodents: Risk of lymphocytic choriomeningitis virus and Leptospira transmission.

The bold values indicate the high percentage of respondents classified turtles and reptiles as low-risk pets, despite guidelines discourage patients to own these pets.

### Suspicion for zoonoses

Healthcare practitioners were asked whether they actively searched for a zoonosis during the differential diagnosis of fever of unknown origin. A 72.2% (26/36) of the infectious disease specialists stated that they routinely include zoonosis screening among their pet-owning patients, compared to 28.8% (32/111) of other specialists (*p* < 0.001). Twenty-nine percent (43/147) of the respondents had previously treated at least one zoonosis (Supplementary File 3). These professionals more frequently search for zoonosis when studying a transplant recipient with fever who owns pets, compared with those responders who have never treated zoonoses [57% (24/42) vs. 31% (32/103); *p* = 0.03].

**Figure 2:**
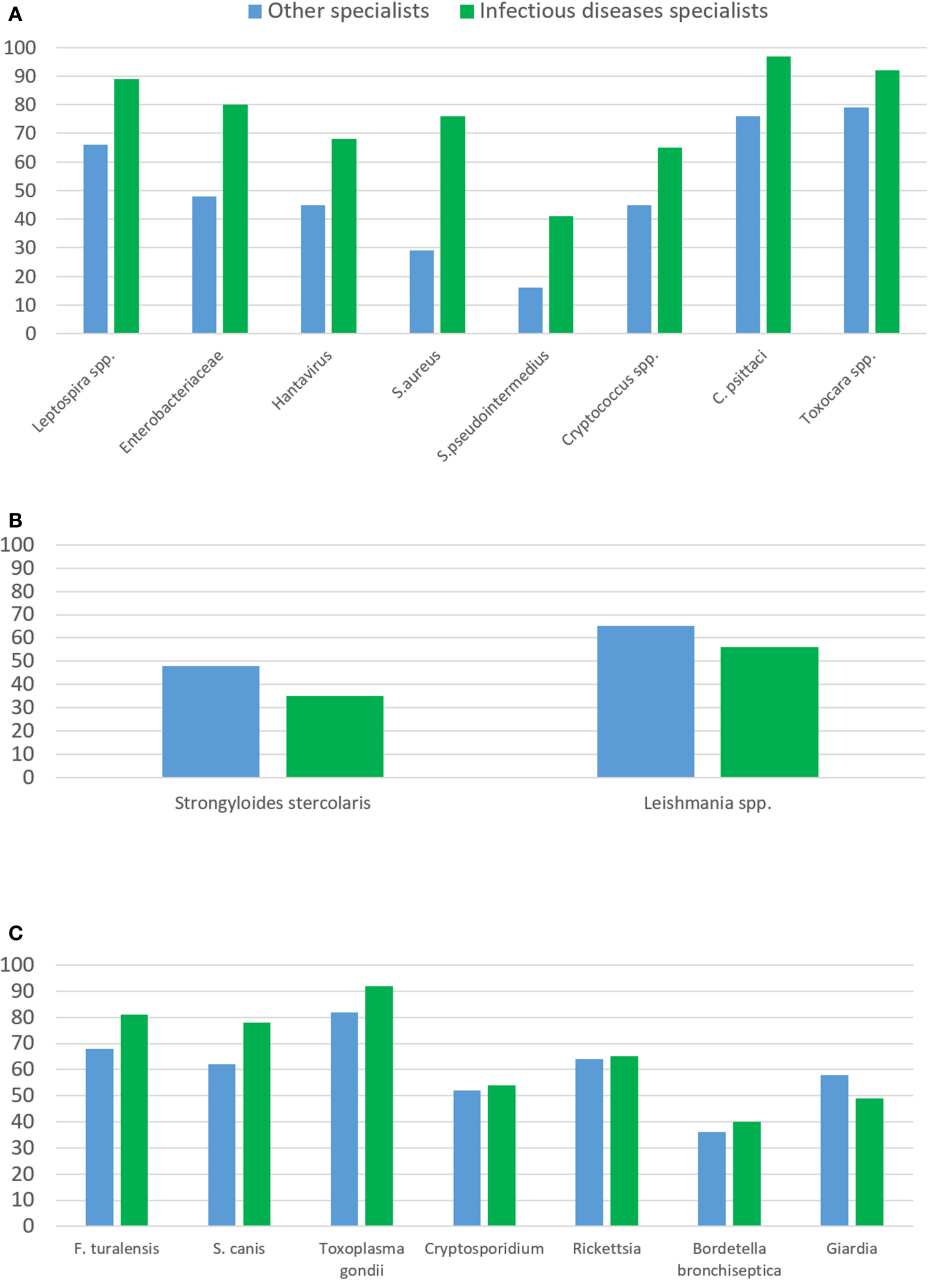
Differences in the identification of pathogens according to the type of medical specialist. **(A)** Zoonotic pathogens significantly identified more frequently by the infectious disease specialists than by the other specialists (*p* < 0.05). Enterobacteriaceae include Campylobacter, *E. coli* and Salmonella. Atypical mycobacteria and Microsporidium canis were also more frequently identified by infectious diseases specialists (57 vs. 76% and 63 vs. 78%, respectively); **(B)** Zoonotic pathogens significantly identified more frequently by the other specialists than by the infectious disease specialists; (**C)** Pathogens with no significant difference in their identification comparing infectious disease specialists with other specialists.

The respondents were asked to select among a list of 23 pathogens that can be transmitted from animals to humans based on literature reports (Figures 2A–C) (13, 16–20). We observed that 52% (12/23) of the pathogens were identified as related to animal contact by more than 70% of the infectious disease specialists, while only 13% (3/23; *Chlamydia psittaci, Toxoplasma gondii* and *Toxocara spp*.) were identified by more than 70% of the remaining specialists (*p* = 0.004). For most pathogens, 20–25% of the respondents were unable to categorize the infection as a zoonosis, 11% (IQR 5–13) for the infectious disease specialists compared with 27% (IQR 24–32) for the other specialists (*p* < 0.001).

Twelve pathogens were identified significantly more by the infectious disease specialists than by the other specialists (Figure 2A); however, two pathogens (*Strongyloides stercoralis* and *Leishmania spp*.) were more readily identified by the other specialists than by the infectious disease specialists (Figure 2B). We analyzed the geographical location of these practitioners, given that most cases of strongyloidiasis and leishmaniasis have been reported in southwestern Europe (21, 22). We observed that, regardless of their specialty, 86% (49/57) of the respondents from Spain, Portugal and Italy could identify *Leishmania* spp. as a possible zoonotic agent, compared to 47% (45/94) of the respondents from other countries (*p* < 0.0001). No significant differences were found for *Strongyloides stercoralis* when comparing respondents from southern Europe (52%; 26/50) and other regions (40.5%; 41/60; *p* = 0.1).

## Discussion

Our results suggest that experienced healthcare practitioners involved in pediatric transplantation do not perceive pets as a potential associated health risk and do not systematically collect information regarding contact with pets. Most centers do not have standardized protocols and official recommendations for animal contact for immunosuppressed patients. The survey reveals a significant variability in clinical practice. Over 30% of the respondents had treated at least one case of zoonosis, however there is a lack of knowledge of the risks associated with the different types of pets and most clinicians do not include zoonosis in the differential diagnosis of fever.

These results likely reflect the lack of evidence-based recommendations, which are mainly based on expert opinion, on anecdotal clinical experiences, extrapolation from experiences on other immunocompromised hosts, and risk warnings arising from case series (9). It is therefore unsurprising that clinical practice varies widely. The recommendations provided by healthcare practitioners are heterogeneous, and usually are based on personal experiences. Practitioners that are pet owners often encourage their patients to have companion animals; hemato-oncologists, infectious disease specialists and practitioners who have treated zoonoses more frequently advise against them. The lack of evidence certainly places practitioners in a complex dilemma when it comes to offer recommendations to patients, however, practitioners should not ignore the risk imposed by pets. A complete anamnesis should always include information regarding animal contact, and the possibility of zoonosis has to be contemplated when evaluating these patients. As there is no evidence to estimate the risk of infection associated to certain pets, there are some recommendations that we can offer to at least minimize the risks, such as a strict veterinary control of the pets, which is included in most guidelines (9, 10).

Several authors highlight that only a few immunocompromised patients are asked about pets or receiving recommendations on safe animal contact (8, 14). However, up to 70% of new pets acquired by families of children with chronic disease are considered as a high infectious risk, either due to the pets age (puppies younger than 6 months) or due to factors related to the animal species (8). Families of immunocompromised children are often unaware about the possibility of acquiring infections from their pets (8). The fact that most healthcare practitioners do not actively include this information in the anamnesis reinforces the idea that pets are not a matter of concern. A recent study that assessed the knowledge and perceptions regarding infectious disease transmission in AAT settings reported that up to 70% of animal handlers working in these programs were not concerned about infectious disease transmission (15). Proper infection control practices by these individuals were lacking despite having undergone animal handler education (15).

The fact that many transmittable infections by pets were not identified as zoonosis by our respondents also explain their low-risk perception. Acute diarrhea caused by *Salmonella* spp. is usually cataloged as a foodborne illness but has been largely reported as a zoonotic infection (23). The suspicion and rate of zoonosis identified by the infectious disease specialists was significantly higher, prompting them to systematically screen for zoonoses during the anamnesis. Multidisciplinary teams including infectious diseases would provide better screening protocols, and promote a prompt detection and treatment of zoonotic infections in immunocompromised children.

A significant percentage of infectious disease specialists were unable to identify pathogens that commonly colonize pets as potentially transmissible to humans, such as *Staphylococcus pseudintermedius, Bordetella bronchiseptica, Cryptococcus neoformans or Mycobacterium tuberculosis/Mycobacterium bovis* (24). *Bordetella bronchiseptica* has been reported to cause pneumonia in transplant recipients after exposure to dogs that have recently been vaccinated with the live-vaccine (25). *C. neoformans* has been found in a high percentage of avian pets' feces and is transmitted through the inhalation of aerosolized organisms from feces (18, 26). Although pets are unlikely to spread *M. bovis*, there are reports of some transmitted cases associated to cats (27).

Transplantation is a complex process that causes high levels of stress for families and patients, as well as increased financial and time burdens, disrupted family interactions, and major restructuring of daily routines. Improved family adjustment following pediatric transplantation has been associated with beneficial medical and psychosocial outcomes (28), and pets could be an additional benefit. Up to 70% of the families of chronically ill children reported that the benefits of pet ownership outweighed the health risks, and 92% of the pet-owning respondents felt that removing their pets would have a negative impact (8). Healthcare practitioners should therefore address this issue with the families, and should communicate both the benefits and risks imposed by pet ownership.

The role of health care professionals in preventing zoonosis is determinant. Expectations and concerns should be discussed with the family. Clinicians should evaluate individual risk based on immunosuppression and local epidemiology. Clear written information should be provided, including timing of new pet acquisition, veterinary and hygiene requirements and additional sources on information. Infectious disease specialists and veterinarians can support the decision making, and need to be involved (29).

Regarding the type of pet, some animals should be avoided such as puppies and kittens younger than 6 months of age, exotic pets, stray and wild animals, reptiles, turtles or amphibians (9, 10). Transplant recipients should avoid cleaning pet litter boxes, bird cages, bird feeders, and fish tanks, disposing of animal waste, or handling animal feces (9, 10). Hands should be washed before and after coming into contact with animals, their food, or supplies. Kissing animals, being licked by animals, bedding with dogs or cats, sharing food with animals, activities with high risk for scratches or bites, or keeping cages or pets' pillows in the patient room should be avoided (9, 10).

On the other hand, routine and proper veterinary care is an essential. The relevance of securing the pet's health should be emphasized by healthcare providers, and individual veterinary procedures should be adjusted (9, 10, 30). Knowledge regarding zoonosis, parasite prevention, dental care or animal feeding should be routinely checked and reinforced. Pet vaccination schedules should be checked (9, 10, 30).

Our study has several limitations, including the missing data, the lack of knowledge about the total number of professionals who initially received the survey, and therefore, the rate of responders, as well as the selection bias inherent to the study design which was based on an online questionnaire. The geographical distribution of the participants is an additional limitation, since most of the clinicians worked in Europe and we report the current knowledge on pet-associated zoonoses and the recommendations provided by pediatric transplantation specialists across 28 different counties.

In summary, our results suggest high heterogeneity and a low-risk perception regarding zoonosis among healthcare professionals that manage pediatric transplant recipients. Further evidence is urgently needed in order to establish evidence-based guidelines for transplant recipients who own pets. In the meantime, health care professionals should address animal contact among other risk factors, ideally from a multidisciplinary approach and with an individual risks-benefits perspective. Health professionals should communicate this information to families in order to improve the quality of life of pediatric transplant recipients.

## Data availability statement

The original contributions presented in the study are included in the article/Supplementary material, further inquiries can be directed to the corresponding author.

## Author contributions

LP: participated in the writing of the initial draft, participated in data analysis, and participated in the performance of the research. PG-S: participated in the performance of the research, data collection, and reviewing the definitive manuscript. TS: participated in research design, participated in the writing of the initial draft, and participated in data analysis. CC: participated in research design, reviewing the definitive manuscript, and participated in data analysis. II, FE, RF, EF, and AP-M: participated in the performance of the research and reviewing the definitive manuscript. AM-E: participated in research design, participated in the writing of the initial draft, participated in data analysis, participated in the performance of the research, and reviewing the definitive manuscript. All authors approved the final manuscript as submitted and agree to be accountable for all aspects of the work.

## Funding

This study was supported by the Health Institute Carlos III, Grant No. PI18CIII/00372 [Carlos III Spanish Health Institute (ISCIII) and Fondos FEDER; EU], by the Spanish Pediatrics Association (AEP) INVEST-AEP 2021 Research Grant and, by the MAPFRE Foundation (Research grants by Ignacio H. de Larramendi 2021). TS has been funded by Carlos III Health Institute-Fondos Feder (BAE21/00022). The funding bodies did not have a role in the design or conduct of the study, the analysis and interpretation of the results, the writing of the report, or the decision to publish.

## Conflict of interest

The authors declare that the research was conducted in the absence of any commercial or financial relationships that could be construed as a potential conflict of interest.

## Publisher's note

All claims expressed in this article are solely those of the authors and do not necessarily represent those of their affiliated organizations, or those of the publisher, the editors and the reviewers. Any product that may be evaluated in this article, or claim that may be made by its manufacturer, is not guaranteed or endorsed by the publisher.

## Supplementary material

Supplementary File 1 Online survey distributed by the European Reference Network TransplantChild and the European Society for Pediatric Infectious Diseases among their members.

Supplementary File 2 Participants' countries and hospitals.

Supplementary File 3 Type of infections related to animal contact previously treated by the 151 respondents.
