# Supplementary material for: Pets for pediatric transplant recipients: To have or not to have
Source: Front Vet Sci. 2022 Sep 8;9:974665. doi: 10.3389/fvets.2022.974665 (PMC9493113; doi:10.3389/fvets.2022.974665)
Supplement: Supplementary File 1 — Online survey distributed by the European Reference Network TransplantChild and the European Society for Pediatric Infectious Diseases among their members. [file Data_Sheet_1.pdf]

FIRST PART: TYPE OF PROFESSIONAL

Gender

- ○ Male
- ○ Female

Age

- ○ <35
- ○ 35-45
- ○ 45-55
- ○ >55

Do you have children?

- ○ Yes
- ○ No

Do you have pets?

- ○ Yes
- ○ No

Your Initials:

In which hospital do you work?

- ○ King's College, London, England
- ○ Vilnius University Hospital Santaros Klinikos, Lithuania
- ○ Centro Hospitalar e Universitário de Coimbra, Portugal
- ○ University Medical Center Utrecht, The Nederlands
- ○ Children's Memorial Health Institute, Poland
- ○ Hospital Universitario la Paz, España
- ○ Medizinische Hochschule Hannover, Germany
- ○ Centro Hospitalar de Lisboa Norte, Portugal
- ○ Ospedale Papa Giovanni XXIII, Bergamo, Italy
- ○ Centro Hospitalar do Porto, Portugal
- ○ Karolinska University Hospital, Sweden
- ○ Assistance Publique Hôpitaux de Paris, Hôpital Bicêtre, France
- ○ Assistance Publique Hôpitaux de Paris, Hôpital Necker Enfants Malades, France
- ○ Skåne University Hospital, Lund, Sweden
- ○ Ospedale Pediatrico Bambino Gesù, Rome, Italy
- ○ University Hospital SaintLuc, Belgium
- ○ Azienda ospedaliera di Padova, Italy
- ○ ISMETT Istituto Mediterraneo per i Trapianti e Terapie ad Alta Specializzazione, Palermo, Italy
- ○ Others

Specify:

Type of Transplant are you involved:

- Renal
- BMT /HSCT
- Cardiac
- Lung
- Liver
- Pancreas
- Intestine
- Multi-organ/multivisceral
- Other

Which is your specialty?

- Pediatric surgeon
- Pediatrician in charge of solid organ transplant recipients
- Pediatrician performing hematopoietic stem-cell transplantation
- Pediatric Infectious Disease specialist
- Others

Specify:

How many children with SOT/HSCT are actually being followed in your Department?

- < 25
- 25-50
- 50-75
- 75-100
- >100

How many years have you been working in pediatric transplantation?

- < 5 years
- 5-10 years
- 10-15 years
- > 15 years

SECOND PART: RECOMMENDATIONS

1. Does your Center/Department have specific written recommendations for transplant patients regarding pets or animal exposures?

- Yes
- No

If the answer is NO, do you actively ask your transplanted patients about pets or animal exposures?

- ○ Yes
- ○ No, I do not routinely ask about pets or animals' exposures to my patients

2. If the patient asks you about buying a pet, what do you usually recommend?

- ○ I recommend NOT buying a pet
- ○ They can buy it, but I remark the importance of a proper veterinarian control of the pet
- ○ I recommend it.
- ○ I encourage them to do it.
- ○ Not know/Not answer

3. If your patient has already a pet, what do you recommend?

- ○ I don't give specific recommendations
- ○ I just recommend contacting their veterinarian
- ○ I give specific recommendations regarding animal exposures, because I believe pets have a beneficial effect
- ○ I advise against having pets

4. Do you know approximately how many of your transplanted patients have animals in their household?

- ○ Yes
- ○ No

5. If you have responded YES, how many of them are pets 'owners?

- ○ < 25%
- ○ 25-50%
- ○ > 50%
- ○ Not answer

6. Is there any initiative in your center involving pets visiting hospitalized children?

- ○ Yes
- ○ No

THIRD PART: KNOWLEDGE ABOUT ZOONOSIS

1. What do you think about the evidence regarding zoonosis in immunocompromised patients?

- ○ There is no good evidence.
- ○ There is some evidence but no enough to influence clinical practice
- ○ There is good evidence and my clinical practice is evidence based
- ○ I am not aware about the evidence

2. Is a pediatric infectious disease specialist usually involved in the management of transplanted children?

- ○ Yes

○ No

3. Do you usually search for zoonosis when studding a transplant recipient with fever who is a pet 'owner?

○ Yes
○ No

4. Have you treated any type of infection transmitted from animals in your transplanted patient?

○ Yes
○ No

Please, if YES, write briefly the type of infection, the type of transplantation and the clinical outcome of your patient.

5. Among the following pets, which ones you believe represent risk of transmission?

	High risk	Low risk	No risk	I don´t know
Dogs	○	○	○	○
Cats	○	○	○	○
Birds	○	○	○	○
Fishes	○	○	○	○
Turtles	○	○	○	○
Reptiles	○	○	○	○
Hamsters / Rabbits	○	○	○	○

6. LAST QUESTION !!!! Which of the following microbes do you believe could be transmitted to your patients from a pet/animals?

Select all you considered.

The question wants to measure your actual knowledge regarding zoonosis. Please, try to answer quickly.

	YES	NO	I DON´T KNOW
Atypical mycobacteria	○	○	○
Aspergillus	○	○	○
Bordetella	○	○	○
Campylobacter	○	○	○
Cryptococcus	○	○	○
Cryptosporidium	○	○	○

C. psittaci	◯	◯	◯
Dermatophytosis	◯	◯	◯
E. Coli	◯	◯	◯
Giardia	◯	◯	◯
Hantavirus	◯	◯	◯
Leptospirosis	◯	◯	◯
Leishmania	◯	◯	◯
S.aureus	◯	◯	◯
Rickettsia	◯	◯	◯
Salmonella	◯	◯	◯
Strongyloides	◯	◯	◯
S. pseudointermedius	◯	◯	◯
S. canis	◯	◯	◯
Tuberculosis	◯	◯	◯
Tularemia	◯	◯	◯
Toxocara	◯	◯	◯
Toxoplasma	◯	◯	◯
