# Supplementary material for: Pets for pediatric transplant recipients: To have or not to have
Source: Front Vet Sci. 2022 Sep 8;9:974665. doi: 10.3389/fvets.2022.974665 (PMC9493113; doi:10.3389/fvets.2022.974665)
Supplement: Supplementary File 2 — Participants' countries and hospitals. [file Data_Sheet_2.docx]

***Supplementary File 2.*** *Participants’* *countries and hospitals*

| **PARTICIPANTS’ COUNTRIES** | **N** | **%** |
| --- | --- | --- |
| **EUROPE** | **106** | **70.2%** |
| Spain | 38 | 25.17% |
| Italy | 12 | 7.95% |
| United Kingdom | 10 | 6.62% |
| Portugal | 7 | 4.64% |
| Hungary | 6 | 3.97% |
| France | 6 | 3.97% |
| Poland | 5 | 3.31% |
| Germany | 4 | 2.65% |
| Belgium | 3 | 1.99% |
| The Netherlands | 2 | 1.32% |
| Lithuania | 2 | 1.32% |
| Slovenia | 2 | 1.32% |
| Sweden | 2 | 1.32% |
| Switzerland | 2 | 1.32% |
| Malta | 1 | 0.66% |
| Ireland | 1 | 0.66% |
| Denmark | 1 | 0.66% |
| Greece | 1 | 0.66% |
| Slovakia | 1 | 0.66% |
| **NORTH AMERICA** | **17** | 11.2% |
| USA | 12 | 7.9% |
| Canada | 5 | 3.31% |
| **SOUTH AMERICA** | **5** | 3.31% |
| Brazil | 4 | 2.65% |
| Chile | 1 | 0.66% |
| **ASIA** | **2** | 1.32% |
| India | 1 | 0.66% |
| United Arab Emirates | 1 | 0.66% |
| **AFRICA** | **2** | 1.32% |
| Egypt | 1 | 0.66% |
| Ivory Coast | 1 | 0.66% |
| **OCEANIA** | **2** | 1.32% |
| Australia | 2 | 1.32% |
| **Non-responders** | **17** | **11.26%** |
| **TOTAL** | **151** | **100%** |

| **PARTICIPANTS’ HOSPITALS** | **N** |
| --- | --- |
| **SPAIN** | |
| Hospital Universitario La Paz (Madrid) | 15 |
| Hospital Universitari Vall d’Hebron (Barcelona) | 5 |
| Hospital Universitario Virgen del Rocio (Seville) | 5 |
| Hospital Infantil Universitario Niño Jesús (Madrid) | 3 |
| Hospital Sant Joan de Déu (Barcelona) | 3 |
| Hospital MD Anderson Cancer Center (Madrid) | 1 |
| Hospital General Universitario Gregorio Marañón (Madrid) | 1 |
| Hospital Universitario Reina Sofía (Córdoba) | 1 |
| Hospital Universitari i Politècnic La Fe (Valencia) | 1 |
| Hospital Universitario Marqués de Valdecilla (Santander) | 1 |
| Hospital Clínico Universitario Virgen de la Arrixaca (Murcia) | 1 |
| Hospital de la Santa Creu i Sant Pau (Barcelona) | 1 |
| **ITALY** | |
| Azienda ospedaliera di Padova (Padova) | 4 |
| Policlinico S. Orsola-Malpighi (Bologna) | 3 |
| IRCCS Istituto Giannina Gaslini (Genova) | 1 |
| Istituto Nazionale dei Tumori (Milan) | 1 |
| Azienda Ospedaliera-Universitaria di Modena (Modena) | 1 |
| Azienda Ospedaliero Universitaria Consorziale Policlinico (Bari) | 1 |
| Ospedale Papa Giovanni XXIII (Bergamo) | 1 |
| **UNITED KINGDOM** | |
| Great Ormond Street Hospital (London) | 3 |
| Imperial College Healthcare NHS Trust (London) | 1 |
| Royal Victoria Infirmary – Newcastle Hospitals (Newcastle) | 1 |
| Cambridge University Hospitals (Cambridge) | 1 |
| St George’s University Hospitals NHS Foundation Trust (London) | 1 |
| Birmingham Women’s and Children’s Hospital (Birmingham) | 1 |
| King’s College Hospital (London) | 1 |
| Great North Children’s Hospital (Newcastle upon Tyne) | 1 |
| **PORTUGAL** | |
| Centro Hospitalar de Lisboa Norte (Lisbon) | 3 |
| Instituto Português de Oncologia de Lisboa Francisco Gentil (Lisbon) | 2 |
| Centro Hospitalar e Universitário de Coimbra (Coimbra) | 1 |
| Centro Hospitalar do Porto (Porto) | 1 |
| **HUNGARY** | |
| South-Pest Hospital Centre – National Institute for Infectology and Haematology (Budapest) | 6 |
| **FRANCE** | |
| Hôpital Necker, Enfants Malades, Assistance Publique Hôpitaux de Paris (Paris) | 4 |
| Hôpital Bicêtre, Assistance Publique Hôpitaux de Paris (Paris) | 2 |
| **POLAND** | |
| Children’s Memorial Health Institute (Warsaw) | 4 |
| University Clinical Hospital (Wroclaw) | 1 |
| **GERMANY** | |
| Medizinische Hochschule Hannover (Hannover) | 3 |
| Universitätsklinikum Düsseldorf (Düsseldorf) | 1 |
| **BELGIUM** | |
| Ghent University Hospital (Ghent) | 1 |
| Cliniques Universitaires Saint-Luc (Brussels) | 1 |
| Universitair Ziekenhuis Leuven (Leuven) | 1 |
| **THE NETHERLANDS** | |
| University Medical Center Utrecht (Utrecht) | 2 |
| **LITHUANIA** | |
| Vilnius University Hospital Santaros Klinikos (Vilnius) | 2 |
| **SLOVENIA** | |
| University Medical Centre Ljubljana (Ljubljana) | 2 |
| **SWEDEN** | |
| Sahlgrenska University Hospital (Gothenburg) | 1 |
| Karolinska University Hospital (Solna) | 1 |
| **SWITZERLAND** | |
| Hôpital du Jura, site de Delémont (Delémont) | 1 |
| Hôpitaux Universitaires de Genève (Geneva) | 1 |
| **MALTA** | |
| Mater Dei Hospital (Msida) | 1 |
| **IRELAND** | |
| National Maternity Hospital, Holles Street (Dublin) | 1 |
| **DENMARK** | |
| Copenhagen University Hospital Rigshospitalet (Copenhagen) | 1 |
| **GREECE** | |
| General University Hospital of Patras (Patras) | 1 |
| **SLOVAKIA** | |
| National Institute of Children’s Diseases (Bratislava) | 1 |
| **UNITED STATES OF AMERICA** | |
| Nationwide Children’s Hospital (Columbus) | 2 |
| University of California, San Francisco Medical Center (San Francisco) | 2 |
| University Health System (San Antonio) | 1 |
| Cleveland Clinic (Cleveland) | 1 |
| Texas Children’s Hospital (Houston) | 1 |
| Mayo Clinic (Rochester) | 1 |
| The Medical University of South Carolina (Mt Pleasant) | 1 |
| Children’s Hospital Colorado (Aurora) | 1 |
| Monroe Carell Jr. Children’s Hospital at Vanderbilt (Nashville) | 1 |
| Dell Children’s Medical Center (Austin) | 1 |
| **CANADA** | |
| Hospital for Sick Children (Toronto) | 2 |
| Centre Hospitalier Universitaire Sainte-Justine (Montreal) | 1 |
| Stollery Children’s Hospital (Edmonton) | 1 |
| University of Manitoba (Winnipeg) | 1 |
| **BRAZIL** | |
| Instituto de Oncologia Pediátrica (São Paulo) | 1 |
| Beneficência Portuguesa de São Paulo (São Paulo) | 1 |
| Universidade Federal de São Paulo (São Paulo) | 1 |
| Hospital Santa Casa de São Paulo (São Paulo) | 1 |
| **CHILE** | |
| Hospital Clínico Pontificia Universidad Católica de Chile (Santiago) | 1 |
| **INDIA** | |
| All India Institute of Medical Sciences, Jodhpur (Jodhpur, India) | 1 |
| **UNITED ARAB EMIRATES** | |
| Sheikh Khalifa Medical City (Abu Dhabi, United Arab Emirates) |  |
| **EGYPT** | |
| Mansoura University Children Hospital (Mansoura, Egypt) | 1 |
| **IVORY COAST** | |
| Centre Hospitalier Universitaire de Angré (Abidjan, Ivory Coast) | 1 |
| **AUSTRALIA** | |
| The Children’s Hospital at Westmead (Sydney, Australia) |  |
| Women’s and Children’s Hospital, Adelaide (Adelaide, Australia) |  |
| **Non-responders** | **17** |
| **TOTAL** | **151** |
