# Supplementary material for: Pets for pediatric transplant recipients: To have or not to have
Source: Front Vet Sci. 2022 Sep 8;9:974665. doi: 10.3389/fvets.2022.974665 (PMC9493113; doi:10.3389/fvets.2022.974665)
Supplement: Supplementary File 3 — Type of infections related to animal contact previously treated by the 151 respondents. [file Data_Sheet_3.docx]

**Supplementary file 3**. Type of infections related to animal contact previously treated by the 151 respondents

| **ZOONOSES** | **N** | **%*** |
| --- | --- | --- |
| Toxoplasmosis | 13 | 8.6% |
| Salmonellosis | 8 | 5.3% |
| Infection due to *Cryptosporidium spp*. | 5 | 3.3% |
| Cat-scratch fever | 5 | 3.3% |
| Leishmaniasis | 4 | 2.65% |
| Infection due to *Cryptococcus neoformans* | 4 | 2.65% |
| Infection due to atypical mycobacteria (1 *M. avium/ 1 M. marinum*) | 2 | 1.32% |
| Toxocariasis | 2 | 1.32% |
| Rabies | 1 | 0.66% |
| Brucellosis | 1 | 0.66% |
| Infection due to *Campylobacter spp.* | 1 | 0.66% |
| Peritonitis due to *Pasteurella multocida* | 1 | 0.66% |
| Disseminated fatal infection due to *Microsporum spp.* | 1 | 0.66% |
| Q fever | 1 | 0.66% |
| Hepatitis E related to animal contact | 1 | 0.66% |
| Infection due to *Bordetella bronchiseptica* | 1 | 0.66% |
| Aspergillosis related to animal contact | 1 | 0.66% |
| Giardiasis | 1 | 0.66% |
| Psittacosis | 1 | 0.66% |
| Echinococcosis | 1 | 0.66% |
| Cysticercosis | 1 | 0.66% |
| Skin infection related to animal contact | 1 | 0.66% |
| Fish-tank associated mycosis | 1 | 0.66% |
| Mycosis related to animal contact | 1 | 0.66% |

**Percentage of respondents who reported having treated each zoonosis*
